# Additional Adverse Perinatal Outcomes With No Effect on Neonatal Mortality and Birth Defects in Pregnancies Conceived by Assisted Reproductive Technology

**DOI:** 10.3389/fped.2022.809259

**Published:** 2022-03-24

**Authors:** Yingying Xiong, Xiaodong Zang, Tingting Xie, Chaolei Yang, Xiaohua Jiang, Mingwu Chen

**Affiliations:** ^1^Department of Pediatrics, The First Affiliated Hospital of Science and Technology of China (USTC), Division of Life Sciences and Medicine, University of Science and Technology of China, Hefei, China; ^2^Department of Pediatrics, Anhui Provincial Hospital, Wannan Medical College, Hefei, China; ^3^Reproductive and Genetic Branch, The First Affiliated Hospital of Science and Technology of China (USTC), Division of Life Sciences and Medicine, University of Science and Technology of China, Hefei, China

**Keywords:** assisted reproductive technology, spontaneous conception, neonatal intensive care unit, perinatal period, adverse outcome

## Abstract

**Background:**

Pregnancy outcomes and perinatal diseases of children conceived by assisted reproductive technology (ART) and spontaneous conception (SC) are still unclear. We sought to compare the effects of ART and SC on adverse neonatal outcomes.

**Methods:**

We included 5,913 neonates admitted to the neonatal intensive care unit (NICU) of the First Affiliated Hospital of the University of Science and Technology of China between January 2017 and December 2020. There were 1,112 (18.8%) ART pregnancies and 4,801 (81.2%) SC pregnancies. Data on maternal characteristics, comorbidities during pregnancy, and neonatal outcomes were collected and analyzed. Logistic regression models estimated the odds ratios (ORs) and 99% CIs of neonatal outcomes according to ART pregnancy. Neonatal outcomes primarily included neonatal respiratory distress syndrome (NRDS), bronchopulmonary dysplasia (BPD), retinopathy of prematurity (ROP), neonatal anemia, birth defects, and mortality.

**Results:**

Among 5,913 neonates, 485 (8.2%) had NRDS, 165 (2.8%) had BPD, 113 (1.9%) had ROP, 602 (10.2%) had neonatal anemia, and 1,112 (18.8%) were ART infants. The incidence of pregnancy-related complications, such as gestational diabetes mellitus (GDM), gestational hypothyroidism, and rheumatic immune diseases, in mothers receiving ART, was higher than that in the SC group. On multivariate analysis, ART was independently associated with NRDS (OR = 1.46; 95% CI, 1.11–1.93; *p* = 0.008) and ROP (OR = 1.79; 95% CI, 1.06–3.05; *p* = 0.031). Moreover, the association persisted after adjustment for maternal age, history of cesarean section, preconception factors, and pregnancy complications. For BPD (OR = 1.44; 95% CI, 0.91–2.27; *p* = 0.117) and neonatal anemia (OR = 1.12; 95% CI, 0.87–1.45; *p* = 0.373), the associations were attenuated substantially when adjusting for pregnancy complications. ART was associated with neither birth defects (OR = 0.98; 95% CI, 0.77–1.25; *p* = 0.889) nor mortality (OR = 0.98; 95% CI, 0.51–1.91; *p* = 0.961).

**Conclusion:**

ART was independently associated with adverse neonatal outcomes, including NRDS and ROP. Therefore, women who conceive by ART must improve their perinatal health and management of pregnancy-related comorbidities to enhance the quality of life of their offspring.

## Introduction

Assisted reproductive technology (ART) has been used in clinical settings for more than 40 years, and the number of babies conceived by ART accounts for ~1% of the total number of births in China (1–4). It is well-known that ART is fundamentally different from natural conception. It involves a series of unnatural methods, such as ovulation induction and *in vitro* fertilization, and may be accompanied by a series of complications, such as ovarian hyperstimulation syndrome (5). Despite the success of ART in overcoming infertility, there are growing concerns regarding its safety and effects on maternal and child health (6). However, scholars have differing opinions on the different maternal and infant outcomes of ART and spontaneous conception (SC). For example, some studies have assumed that preterm birth is more prevalent in pregnancies conceived through ART, and the incidence of preterm delivery (<34 weeks) associated with ART is significantly higher than that of SC (7). A population-based study in Japan found that couples experiencing infertility had an increased risk of adverse maternal and perinatal outcomes (8). However, Jiang et al. found that ART did not increase the risk of preterm birth in a comparison of neonatal outcomes of twin pregnancies conceived by ART and SC (9). However, little is known about the relationship between ART and adverse neonatal outcomes. Thus, a systematic analysis of the impact of ART on perinatal outcomes should be performed to further direct perinatal healthcare and management of pregnancy-related comorbidities.

In this study, we comprehensively compared maternal and infant perinatal indices based on the different conception methods. We enrolled all newborns admitted to the neonatal intensive care unit (NICU) of the First Affiliated Hospital of the University of Science and Technology of China (USTC) between January 2017 and December 2020, and we analyzed the maternal characteristics, pregnancy-related comorbidities, and neonatal outcomes. Although ART pregnancies did not significantly increase neonatal mortality or birth defects, we found that ART was independently associated with adverse perinatal outcomes, including neonatal respiratory distress syndrome (NRDS) and retinopathy of prematurity (ROP).

## Materials and Methods

### Study Population

All newborns admitted to the NICU of the First Affiliated Hospital of the USTC between January 2017 and December 2020 were enrolled in this case–control study. Newborns were divided into ART and SC groups according to the conception method.

The inclusion criteria were as follows: (1) all newborns in the ART group who were born after ART treatment to alleviate tubal blockage, ovulation disorders, *in vivo* fertilization disorders, and so on; (2) all newborns in the control group who were born from natural pregnancies; (3) newborns admitted to the NICU within 7 days after birth for treatment; and (4) parturients who never used drugs that could affect fetal growth and development.

**Figure 1:**
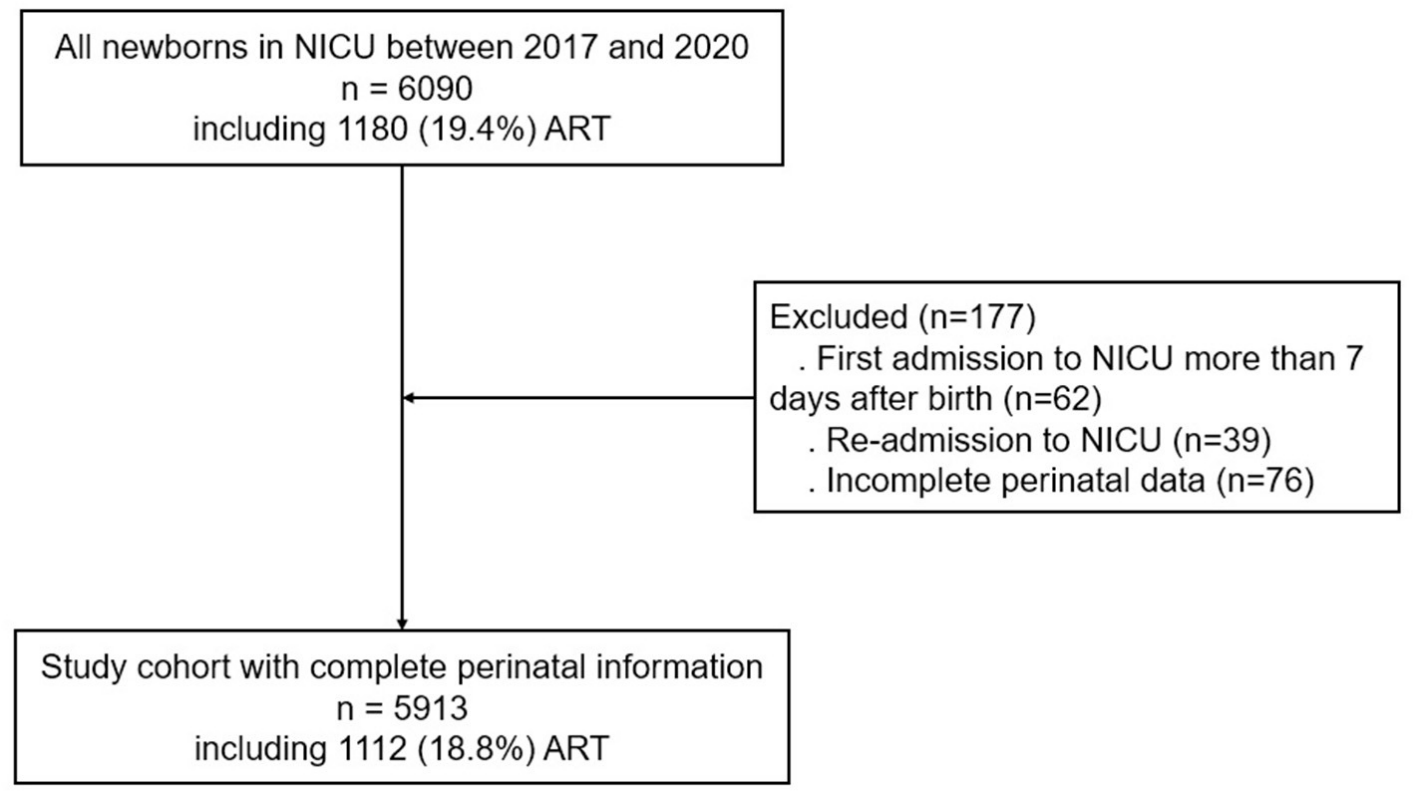
Clinical pathway map of research object selection.

The exclusion criteria were as follows: (1) newborns who were secondarily admitted to the hospital during the neonatal period and (2) newborns with incomplete perinatal data (Figure 1).

The study was reviewed and approved by the Ethics Committee of the First Affiliated Hospital of the USTC (registration number: 2021-RH-104).

### Data Collection for the Perinatal Period

Parturient information, including maternal age, pregnancy comorbidities, mode of delivery, placenta, amniotic fluid, and fetal membranes during delivery, newborn information (such as sex, gestational age at birth, birth weight, body length, and head circumference), and Apgar scores at 1 and 5 min were collected. All indices collected were from the inpatient medical records of the mothers and infants.

### Effectiveness Evaluation Index and Safety Evaluation Index of Assisted Reproductive Technology

All maternal pregnancy complications, including gestational diabetes mellitus (GDM), pregnancy-induced hypertension (PIH), intrahepatic cholestasis of pregnancy (ICP), gestational anemia, gestational hypothyroidism, and rheumatic immune disease, were recorded.

The perinatal complications of newborns and specific neonatal diseases, including NRDS, bronchopulmonary dysplasia (BPD), ROP, necrotizing enterocolitis (NEC), neonatal anemia, neonatal brain injury, intracranial hemorrhage, atrial septal defect, ventricular septal defect, neonatal asphyxia, and septicemia, were recorded.

### Statistical Analysis

SPSS statistical software (version 25.0) was used to input and analyze the data. Maternal and child characteristics are presented as number of cases (*n*) and percentage (%) for categorical data. The groups were compared using the chi-squared test and Fisher's exact test for categorical variables. Continuous data are expressed as the mean ± SD (χ ± s). The Kolmogorov–Smirnov test was used to confirm the normality of the data, and the Mann–Whitney test was used to compare two continuous variables that were not normally distributed. Multiple logistic regression analysis was used to adjust for potential confounding factors. We estimated the odds ratios (ORs) and 95% CIs of neonatal outcomes according to ART pregnancy using multivariable logistic regression, adjusting for maternal age, history of cesarean section, preconception factors, and pregnancy complications. Statistical significance was set at *p* < 0.05.

## Results

### Maternal Characteristics Between the Spontaneous Conception and Assisted Reproductive Technology Groups

From January 2017 to December 2020, 5,913 newborns were enrolled in this study, including 1,112 (18.8%) in the ART group and 4,801 (81.2%) in the SC group. The average maternal age in the ART group (31.95 ± 4.47 years) was 31.95 years, which was higher than that in the SC group (30.40 ± 4.93 years) (*p* < 0.001). Compared with mothers who conceived by SC, mothers who conceived by ART were more likely to have advanced maternal age (SC vs. ART: 16.3 vs. 19.3%; *p* = 0.014) or be primiparous (SC vs. ART: 33.6% vs. 49.3%; *p* < 0.001). Interestingly, there were significantly more multiparous women in the ART group with a poor pregnancy history (SC vs. ART: 13.8 vs. 21.5%; *p* < 0.001), and they were more likely to choose cesarean delivery (SC vs. ART: 68.8 vs. 90.3%; *p* < 0.001) (Table 1).

**Table 1:** Maternal characteristics between the SC and ART groups.

**Variables**	**Overall (%)**	**SC (%)**	**ART (%)**	***p*** **-value**
Number of cases	*N* = 5,913	*N* = 4,801 (81.2)	*N* = 1,112 (18.8)	–
Maternal age (years)	30.69 ± 4.89	30.40 ± 4.93	31.95 ± 4.47	<0.001**
Advanced maternal age	996 (16.8)	781 (16.3)	215 (19.3)	0.014*
Primiparous women	2,163 (36.6)	1,615 (33.6)	548 (49.3)	<0.001*
Adverse pregnancy history	903 (15.3)	664 (13.8)	239 (21.5)	<0.001*
Previous cesarean section	655 (11.1)	611 (12.7)	44 (4.0)	<0.001*
Reproductive system diseases	216 (3.7)	141 (2.9)	75 (6.7)	<0.001*
Pregestational thyroid dysfunction	95 (1.6)	78 (1.6)	17 (1.5)	0.819*
Pregestational autoimmune diseases	95 (1.6)	77 (1.6)	18 (1.6)	0.972*

*Continuous variables are represented by mean ± SD ($\bar{\text{x}}$ ± s). Categorical variables are represented by the proportion and percentage of the total*.

*ART, assisted reproductive technology; SC, spontaneous conception*.

* *Chi-square test*.

** *Mann–Whitney test*.

### Maternal Pregnancy Complications Between the Spontaneous Conception and Assisted Reproductive Technology Groups

Compared to the mothers who conceived by SC, those who conceived by ART were more likely to have GDM (SC vs. ART: 14.2 vs. 17.3%; *p* = 0.009), gestational hypothyroidism (SC vs. ART: 6.9 vs. 12.7%; *p* < 0.001), and rheumatic immune diseases (SC vs. ART: 2.3 vs. 3.8%; *p* = 0.004). Furthermore, there were no significant differences between the two groups in terms of newborn PIH (SC vs. ART: 14.7 vs. 14.2%; *p* = 0.660) or ICP (SC vs. ART: 3.7 vs. 4.1%; *p* = 0.521) (Table 2).

**Table 2:** Maternal pregnancy complications between the SC and ART groups.

**Variables**	**Overall (%)**	**SC (%)**	**ART (%)**	***p*** **-value**
Number of cases	*N* = 5,913	*N* = 4,801 (81.2)	*N* = 1,112 (18.8)	–
Placental abnormalities	1,303 (22.0)	1,092 (22.7)	211 (19.0)	0.006*
Amniotic fluid abnormalities	948 (16.0)	853 (17.8)	95 (8.5)	<0.001*
PROM	1,128 (19.1)	942 (19.6)	186 (16.7)	0.027*
PIH	865 (14.6)	707 (14.7)	158 (14.2)	0.660*
GDM	872 (14.7)	680 (14.2)	192 (17.3)	0.009*
ICP	225 (3.8)	179 (3.7)	46 (4.1)	0.521*
Gestational anemia	225 (3.8)	194 (4.0)	31 (2.8)	0.049*
Thrombocytopenia in pregnancy	155 (2.6)	136 (2.8)	19 (1.7)	0.035*
Gestational hypothyroidism	470 (7.9)	329 (6.9)	141 (12.7)	<0.001*
Rheumatic immune disease	151 (2.6)	109 (2.3)	42 (3.8)	0.004*
Pregnancy infection	110 (1.9)	96 (2.0)	14 (1.3)	0.100*
Cesarean section	4,305 (72.8)	3,301 (68.8)	1,004 (90.3)	<0.001*

*Categorical variables are expressed as proportions and percentages of the total*.

*ART, assisted reproductive technology; SC, spontaneous conception; PROM, premature rupture of membranes; PIH, pregnancy-induced hypertension; GDM, gestational diabetes mellitus; ICP, intrahepatic cholestasis of pregnancy*.

* *Chi-square test*.

### Neonatal Outcomes Between the Spontaneous Conception and Assisted Reproductive Technology Groups

For newborns, multiple births (SC vs. ART: 13.6 vs. 70.2%; *p* < 0.001) were more common in the ART group. The gestational age (SC vs. ART: 253.06 ± 21.46 vs. 245.45 ± 18.68 days; *p* < 0.001), birth weight (SC vs. ART: 2,645.08 ± 785.18 vs. 2,377.84 ± 641.07 g; *p* < 0.001), length (SC vs. ART: 47.05 ± 10.91 vs. 47.01 ± 24.33 cm; *p* < 0.001), and head circumference (SC vs. ART: 32.19 ± 2.70 vs. 31.73 ± 2.60 cm; *p* < 0.001) of newborns in the ART group were lower than those in the SC group. Specifically, newborns with gestational age of <34 weeks (SC vs. ART: 20.4 vs. 23.2%; *p* = 0.038) were more common in the ART group.

Regarding neonatal diseases, there was a greater likelihood of BPD (SC vs. ART: 2.6 vs. 3.7%; *p* = 0.044), NRDS (SC vs. ART: 7.8 vs. 9.8%; *p* = 0.031), ROP (SC vs. ART: 1.7 vs. 2.8%; *p* = 0.018), and neonatal anemia (SC vs. ART: 9.8 vs. 12.0%; *p* = 0.029) in the ART group. Furthermore, there were no significant differences between the two groups in terms of newborn birth defects (SC vs. ART: 12.0 vs. 12.3%; *p* = 0.781) or mortality (SC vs. ART: 1.5 vs. 1.4%; *p* = 0.801) (Table 3).

**Table 3:** Neonatal outcomes between the SC and ART groups.

**Variables**	**Overall (%)**	**SC (%)**	**ART (%)**	***p*** **-value**
Number of cases	*N* = 5,913	*N* = 4,801 (81.2)	*N* = 1,112 (18.8)	–
Male	3,338 (56.5)	2,717 (56.6)	621 (55.8)	0.651*
Female	2,575 (43.5)	2,084 (43.4)	491 (44.2)	–
Multiple births	1,434 (24.3)	653 (13.6)	781 (70.2)	<0.001*
Gestational age <34 weeks	1,237 (20.9)	979 (20.4)	258 (23.2)	0.038*
Gestational age (day)	251.63 ± 21.17	253.06 ± 21.46	245.45 ± 18.68	<0.001**
Birth weight (g)	2,594.82 ± 767.26	2,645.08 ± 785.18	2,377.84 ± 641.07	<0.001**
Length at birth (cm)	47.04 ± 14.42	47.05 ± 10.91	47.01 ± 24.33	<0.001**
Head circumference at birth (cm)	32.10 ± 2.69	32.19 ± 2.70	31.73 ± 2.60	<0.001**
1-min Apgar score	8.62 ± 1.89	8.62 ± 1.87	8.63 ± 1.95	0.903**
5-min Apgar score	9.33 ± 1.28	9.33 ± 1.25	9.32 ± 1.40	0.139**
BPD	165 (2.8)	124 (2.6)	41 (3.7)	0.044*
NRDS	485 (8.2)	376 (7.8)	109 (9.8)	0.031*
NEC	60 (1.0)	45 (0.9)	15 (1.3)	0.217*
ROP	113 (1.9)	82 (1.7)	31 (2.8)	0.018*
Neonatal brain injury	2,118 (35.8)	1,729 (36.0)	389 (35.0)	0.518*
Intracranial hemorrhage	312 (5.3)	248 (5.2)	64 (5.8)	0.428*
Atrial septal defect	397 (6.7)	316 (6.6)	81 (7.3)	0.399*
Ventricular septal defect	82 (1.4)	65 (1.4)	17 (1.5)	0.653*
Neonatal asphyxia	897 (15.2)	736 (15.3)	161 (14.5)	0.476*
Neonatal pneumonia	382 (6.5)	319 (6.6)	63 (5.7)	0.231*
Septicemia	120 (2.0)	102 (2.1)	18 (1.6)	0.281*
Neonatal cholestasis	133 (2.2)	115 (2.4)	18 (1.6)	0.116*
Abnormal liver function	52 (0.9)	45 (0.9)	7 (0.6)	0.322*
Thyroid dysfunction	78 (1.3)	68 (1.4)	10 (0.9)	0.173*
Abnormal coagulation function	574 (9.7)	452 (9.4)	122 (11.0)	0.114*
Neonatal anemia	602 (10.2)	469 (9.8)	133 (12.0)	0.029*
Birth defects	714 (12.1)	577 (12.0)	137 (12.3)	0.781*
Mortality	90 (1.5)	74 (1.5)	16 (1.4)	0.801*

*Categorical variables are expressed as proportions and percentages of the total*.

*ART, assisted reproductive technology; SC, spontaneous conception; BPD, bronchopulmonary dysplasia; NRDS, neonatal respiratory distress syndrome; NEC, necrotizing enterocolitis; ROP, retinopathy of prematurity*.

* *Chi-square test*.

** *Mann–Whitney test*.

### Association of Assisted Reproductive Technology With Adverse Neonatal Outcomes

Among 5,913 neonates, 485 (8.2%) had NRDS, 165 (2.8%) had BPD, 113 (1.9%) had ROP, 602 (10.2%) had neonatal anemia, and 1,112 (18.8%) were ART infants. We composed a variety of logistic regression analysis models in which NRDS, BPD, ROP, neonatal anemia, birth defects, and mortality served as dependent variables and ART served as an independent variable. To avoid the influence of confounding factors, the data were further adjusted for maternal age, previous cesarean section, preconception factors, and pregnancy complications (GDM, hyperthyroidism, hypothyroidism, and autoimmune diseases) in the multiple regression analysis. On multivariate analysis, ART was independently associated with NRDS (OR = 1.46; 95% CI, 1.11–1.93; *p* = 0.008) and ROP (OR = 1.79; 95% CI, 1.06–3.05; *p* = 0.031), and the association persisted after adjustment for confounders. For BPD (OR = 1.44; 95% CI, 0.91–2.27; *p* = 0.117) and neonatal anemia (OR = 1.12; 95% CI, 0.87–1.45; *p* = 0.373), the associations were attenuated substantially when adjusting for pregnancy complications. ART was not independently associated with birth defects (OR = 0.98; 95% CI, 0.77–1.25; p = 0.889) or mortality (OR = 0.98; 95% CI, 0.51–1.91; *p* = 0.961) (Table 4).

**Table 4:** Association of ART with adverse neonatal outcomes.

**Variable**	**SC, OR (95% CI)**	**ART, OR (95% CI)**	***p*** **-value**
Number of participants	4,801	1,112	
**NRDS**
Number of cases/total participants	376/4,801 (7.8%)	109/1,112 (9.8%)	
Model 1^a^	1.00 (reference)	1.37 (1.08,1.73)	0.010
Model 2^b^	1.00 (reference)	1.46 (1.11,1.93)	0.008
**BPD**
Number of cases/total participants	124/4,801 (2.6%)	41/1,112 (3.7%)	
Model 1^a^	1.00 (reference)	1.53 (1.04,2.23)	0.029
Model 2^b^	1.00 (reference)	1.44 (0.91,2.27)	0.117
**ROP**
Number of cases/total participants	82/4,801 (1.7%)	31/1,112 (2.8%)	
Model 1^a^	1.00 (reference)	1.65 (1.06,2.56)	0.027
Model 2^b^	1.00 (reference)	1.79 (1.06,3.05)	0.031
**Neonatal anemia**
Number of cases/total participants	469/4,801 (9.8%)	133/1,112 (12.0%)	
Model 1^a^	1.00 (reference)	1.36 (1.09,1.68)	0.006
Model 2^b^	1.00 (reference)	1.12 (0.87,1.45)	0.373
**Birth defects**
Number of cases/total participants	577/4,801 (12.0%)	137/1,112 (12.3%)	
Model 1^a^	1.00 (reference)	0.96 (0.78,1.18)	0.665
Model 2^b^	1.00 (reference)	0.98 (0.77,1.25)	0.889
**Mortality**
Number of cases/total participants	74/4,801 (1.5%)	16/1,112 (1.4%)	
Model 1^a^	1.00 (reference)	0.96 (0.54,1.68)	0.876
Model 2^b^	1.00 (reference)	0.98 (0.51,1.91)	0.961

*ART, assisted reproductive technology; SC, spontaneous conception; BPD, bronchopulmonary dysplasia; NRDS, neonatal respiratory distress syndrome; ROP, retinopathy of prematurity; OR, odds ratio*.

a *Model 1: adjusted for maternal age, advanced maternal age, primiparous women, previous cesarean section, adverse pregnancy history, and prepregnancy reproductive system diseases*.

b *Model 2: model 1 plus multiple pregnancies, cesarean section, placental abnormalities, premature rupture of membranes, amniotic fluid abnormalities, gestational diabetes mellitus, gestational anemia, thrombocytopenia in pregnancy, gestational hypothyroidism, and rheumatic immune disease*.

## Discussion

In this study, we compared the effects of pregnancies conceived by ART and SC on maternal and perinatal outcomes. The incidence of pregnancy complications, such as GDM, gestational hypothyroidism, and rheumatic immune diseases, was higher in the ART group than in the SC group. Among the NICU neonatal population, one of our primary findings was that ART had a significantly positive association with NRDS and ROP. This association persisted after adjusting for preconception and pregnancy comorbidity variables. However, the association of ART with BPD and neonatal anemia was substantially attenuated when adjusting for pregnancy complications.

Our study found that GDM was significantly more common in ART pregnancies, which is consistent with previous findings (3, 10). Progesterone is always used to support the luteal phase during the ART treatment cycle and the first 3 months of pregnancy, but progesterone increases insulin resistance, which may lead to GDM (10, 11). Additionally, ovarian hyperstimulation syndrome, advanced maternal age, and polycystic ovary syndrome are common risk factors for GDM in pregnant women (12, 13). Specifically, rheumatic immune diseases during pregnancy were more common in the ART group than in the SC group. A possible explanation is that antiphospholipid antibodies and other autoantibodies (such as anti-oocytes) are associated with a high risk of infertility, and women with autoimmune diseases experience decreased fertility after repeated miscarriages and are more likely to choose ART to conceive (14, 15). We also observed more thyroid abnormalities associated with autoimmunity. Ovarian stimulation is also related to decreased thyroid function, and pregnant women who conceived through ART often have poor thyroid function (16, 17). Therefore, women with abnormal thyroid function should be provided levothyroxine supplementation before conception, and thyroid function should be monitored regularly during pregnancy.

For the neonatal outcomes, ART was independently associated with NRDS and ROP after adjustment for confounders. In this study, the association of ART with NRDS and ROP was not attenuated after additional adjustment for pregnancy comorbidity variables. This indicates that the association of ART with NRDS and ROP cannot be fully explained by pregnancy comorbidities. NRDS is a common disease in infants, with an incidence rate of 7% (18). Known risk factors for NRDS include GDM, PIH, cesarean delivery, preterm birth, and placenta previa (19–21). Recent studies have found that various environmental and genetic factors can also affect the development of NRDS (22, 23). Our findings show a clear correlation between ART and NRDS. A study by Lin et al. showed no significant differences in NRDS and ventilator support between the ART and SC groups (24). The differences may be associated with population and regional differences or with variations in sample size and research methods. Oxygen exposure, low birth weight, low gestational age, and breathing problems are risk factors for ROP (25, 26). Some studies found that babies conceived by ART were more prone to developing ROP than babies conceived naturally, which seemed to be more severe. Premature birth and genetic abnormalities caused by ART may be risk factors for ROP (27, 28). After preconception factors were adjusted, ART was associated with BPD and neonatal anemia. However, the association of ART with BPD and neonatal anemia was substantially attenuated when adjusting for pregnancy complications. This indicates that the association of ART with BPD and anemia is more strongly mediated by pregnancy complications. Correlation analysis of neonatal diseases in the perinatal period indicates that ART is primarily associated with preterm diseases. Premature delivery is the major factor affecting perinatal outcomes of pregnancies conceived by ART (29). Therefore, to avoid premature delivery, it is necessary to improve the management, detection, and treatment of complications in pregnancies conceived by ART.

Birth defects are closely related to the perinatal period and infant mortality (30). However, there is still no consensus on whether ART increases the incidence of neonatal birth defects. Through a meta-analysis of previous literature, Zhao et al. found that compared with natural conception, newborns conceived by ART have an increased risk of birth defects (31). Others have concluded that chromosomal, genitourinary, and circulatory system malformations are more common in ART conception (32). However, Yan et al. found that the incidence of birth defects in newborns conceived by ART did not increase, which is consistent with our findings (33). The differences in outcome were related to many factors, such as measures of birth defect, adjustment for confounders, and study populations.

ART is associated with an increased risk for adverse perinatal outcomes. One possible explanation is that the operations involved in the ART process may lead to undesirable results. The growth and development of embryos and fetuses are complex processes, and some studies have found that ART is related to abnormal DNA methylation in human gametes, embryos, placenta, and umbilical cord samples, which is largely related to *in vitro* operations (34, 35). Treatment during the ART process may also affect gamete development and increase the susceptibility of children to diseases (36). Currently, the underlying mechanisms involved in the association between ART and adverse neonatal outcomes are unclear. Further investigation is warranted to elucidate the causality and underlying mechanisms.

In this study, the multidimensional observation and analysis of perinatal outcomes related to ART from pregnancy and neonatal outcomes in a large sample size provided a comprehensive understanding of the impact of different conception methods on neonatal outcomes. However, this was a single-center retrospective clinical study based on the NICU, and the conclusions of our study must be proven in more multicenter, large-scale clinical studies.

In summary, ART was independently associated with neonatal outcomes, including NRDS and ROP. More ART parturients had previous reproductive system diseases, GDM, gestational hypothyroidism, or pregnancies complicated by rheumatic immune diseases. Therefore, it is suggested that women who conceive *via* ART must improve perinatal care and management of pregnancy-related complications, maintain physical and mental health and a healthy diet, and receive prenatal care to avoid premature delivery.

## Data Availability Statement

The original contributions presented in the study are included in the article/supplementary material, further inquiries can be directed to the corresponding author/s.

## Ethics Statement

The studies involving human participants were reviewed and approved by Ethics Committee of the First Affiliated Hospital of USTC with registration number 2021-RH-104. Written informed consent to participate in this study was provided by the participants' legal guardian/next of kin.

## Author Contributions

MC and XJ were involved in the study design, execution and analysis, article drafting, and critical discussion. YX, TX, and CY were involved in the data collection. YX, XJ, and MC drafted and reviewed the manuscript. XZ was involved in the critical discussion. All authors approved the final version to be published.

## Funding

The study was supported by the Natural Science Foundation of Anhui Province (grant no. 21608085MH196) and the Ministry of Science and Technology of the People's Republic of China (grant no. 2018YFC1003700).

## Conflict of Interest

The authors declare that the research was conducted in the absence of any commercial or financial relationships that could be construed as a potential conflict of interest.

## Publisher's Note

All claims expressed in this article are solely those of the authors and do not necessarily represent those of their affiliated organizations, or those of the publisher, the editors and the reviewers. Any product that may be evaluated in this article, or claim that may be made by its manufacturer, is not guaranteed or endorsed by the publisher.

## Abbreviations

ART: assisted reproductive technology
SC: spontaneous conception
PROM: premature rupture of membranes
PIH: pregnancy-induced hypertension
GDM: gestational diabetes mellitus
ICP: intrahepatic cholestasis of pregnancy
BPD: bronchopulmonary dysplasia
NRDS: neonatal respiratory distress syndrome
NEC: necrotizing enterocolitis
ROP: retinopathy of prematurity
OR: odds ratio.
